# Natural course of hemoglobin level after total knee arthroplasty and the benefit of tranexamic acid injection in the joint

**DOI:** 10.1097/MD.0000000000027097

**Published:** 2021-09-03

**Authors:** Myung Rae Cho, Chung Mu Jun, Suk Kyoon Song, Won Kee Choi

**Affiliations:** Department of Orthopaedic Surgery, College of Medicine, Daegu Catholic University, Daegu city, Korea.

**Keywords:** hemoglobin, total knee arthroplasty, tranexamic acid, transfusion

## Abstract

We retrospectively investigated the natural course of hemoglobin (Hb) level after total knee arthroplasty (TKA) and identified the benefit of tranexamic acid injection at the operation field for unilateral TKA patients who have not received blood transfusions.

There were 115 cases conducted by a surgeon who performed TKA without injecting tranexamic acid and 62 cases by another surgeon with injection. During 2-weeks of hospitalization, Hb level was checked on the day of surgery and 1, 2, 3, 5, 7, 11 days after surgery.

Regardless of whether whom the operator was and tranexamic acid was injected or not, the same natural course of Hb level after TKA was observed. The lowest value of Hb was shown at postoperative day 3, after which it tended to recover. In repeated measures analysis of variance test, mean difference from preoperative Hb level showed a statistically significant difference between tranexamic acid injected and noninjected groups (*P* = .01). In post hoc test, the differences from preoperative Hb levels were significantly lower at all measurements in surgeon with injection of tranexamic acid.

When deciding whether to transfuse after TKA, it should be noted that the patient tends to show the lowest Hb level on postoperative day 3. Also, the authors emphasize that tranexamic acid injection in the joint at the operation field is an effective method to reduce the loss of Hb after TKA.

## 1 Introduction

According to Park et al,^[1]^ the amount of blood loss in total knee arthroplasty (TKA) can be more than 2 L. The need for blood transfusion following TKA varied widely depending on surgeons (4.8%–63.8%).^[2]^ Surgeons are concerned about blood loss of TKA and recovering hemoglobin (Hb), because blood transfusion after TKA could lead to postoperative infection and other adverse effects.^[3–8]^ Currently, staged bilateral TKA is often conducted during admission period and interval between the first and the second operation is variable at each center. However, there is a lack of studies of whether the patient's Hb level could recover for anesthesia during that period before surgery. In particular, Hb levels in patients could keep decrease several days after TKA due to the hidden blood loss,^[9]^ which include extravasation of blood into the tissues, residual blood in the knee and loss due to hemolysis, thus staged bilateral TKA may increase the need for allogenic blood transfusion.

In this study, we investigated the changes in Hb level during the 2-weeks of hospitalization for unilateral TKA patients who have not received blood transfusions. We also compared the effects of tranexamic acid on Hb levels depending on whether tranexamic acid is injected into the joint during surgery. This trial was approved by the institutional review board (approval number: CR-20-218) of our hospital and conducted in accordance with the declaration of Helsinki.

## 2 Materials and methods

### 2.1 Patients

**Figure 1:**
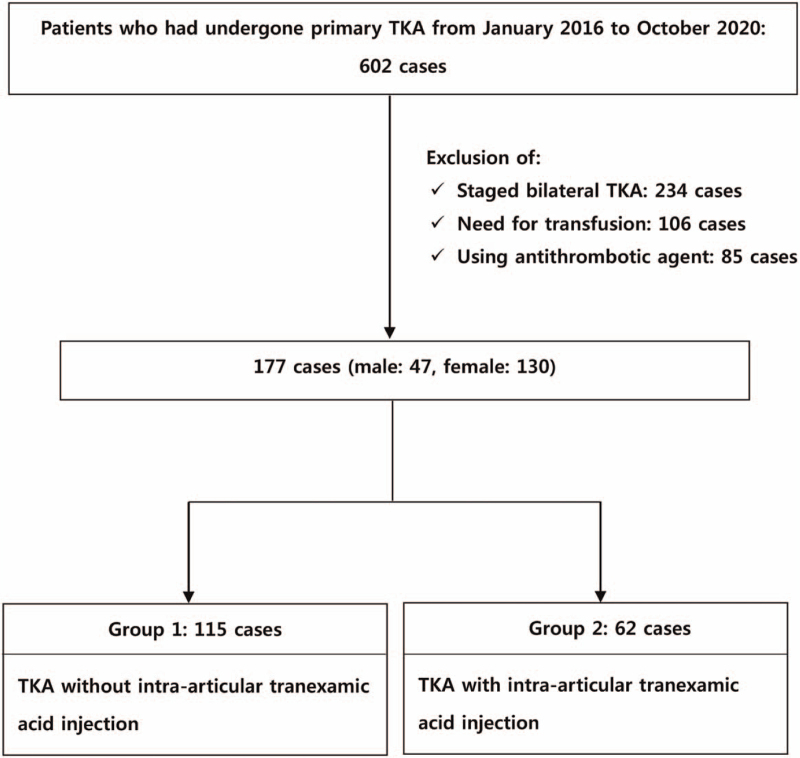
Flow chart of patients. TKA = total knee arthroplasty.

We conducted a retrospective study of patients who had undergone primary TKA for osteoarthritis by 2 skilled orthopedic surgeons in our hospital from January 2016 to October 2020. Among the 602 patients who had undergone primary TKA during this period, 177 patients were eligible for this study. Patients with antithrombotic agent (85 cases) and who underwent staged bilateral TKA (234 cases) or who received transfusion (106 cases) during the admission period were excluded (Fig. 1). The study involved a total of 177 TKA cases, 115 cases conducted by a surgeon who performed TKA without injecting tranexamic acid (group 1), 62 cases by another surgeon who performed TKA injecting tranexamic acid (group 2) (Table 1).

**Table 1:** Epidemiology of all participants.

Variable	Group 1 (N = 115)	Group 2 (N = 62)	Total (N = 117)	*P* value
Age (yr)	70.85 ± 7.47	68.55 ± 8.05	70.05 ± 7.73	.058
Body mass index (m/kg^2^)	26.19 ± 2.83	25.30 ± 3.27	25.88 ± 3.01	.061
Preoperative Hemoglobin	13.23 ± 1.20	12.86 ± 1.25	13.10 ± 1.23	.056
Gender (F/M)	(80/35)	(50/12)	(130/47)	.113

F = female, M = male, N = number. Group 1: navigated total knee arthroplasty without tranexamic acid injection. Group 2: total knee arthroplasty (manual method or navigation method) with tranexamic acid injection.

### 2.2 Surgical techniques

Tourniquets were applied in all operations. Midline skin incision and the medial parapatellar approach were applied in all the cases. Measured gap technique was used for bone resections. Both femoral and tibial components were fixed with bone cement. Posterior cruciate substituting type of implant was used for all TKA. One surgeon who did not injected tranexamic acid performed TKA using navigation for all cases. Another person who injected tranexamic acid into the joint performed TKA using navigation for 24 cases, and manual method for 38 cases. The Imageless Navigation System version 2.6 (BrainLAB, Feldkirchen, Germany) was used in all navigated TKA cases. In the manual TKA cases, the entry point for femoral intramedullary rod was closed by an autologous bone plug. All patients had drainage catheter which was removed at postoperative day (POD) 2 or POD 3 depend on the amount of drainage. The same postoperative rehabilitation protocols for TKA were applied in all patients.

### 2.3 Measurement of hemoglobin level

Both surgeons used the same critical pathway during the patient's admission period, conducting blood tests at the same time. During 2-weeks of hospitalization period, Hb level was checked at the day of surgery and 1, 2, 3, 5, 7, 11 days after surgery. In the case of surgeon who injected tranexamic acid (group 2), the Hb level was checked in the outpatient clinic after 4 weeks from surgery.

### 2.4 Statistical analysis

All analyses were performed with IBM SPSS version 19.0 software (SPSS Inc., Chicago, IL) for Windows. Repeated measures analysis of variance (ANOVA) test was used to identify the changes in Hb levels during the 2-weeks of hospitalization for patients and the effects of tranexamic acid on Hb levels. Post hoc test of the differences in Hb levels in the 2 groups over time was performed using the *t* test. A *P* value less than or equal to .05 was considered to indicate statistical significance.

## 3 Results

### 3.1 The changes in hemoglobin levels during the 2-week hospitalization period

**Figure 2:**
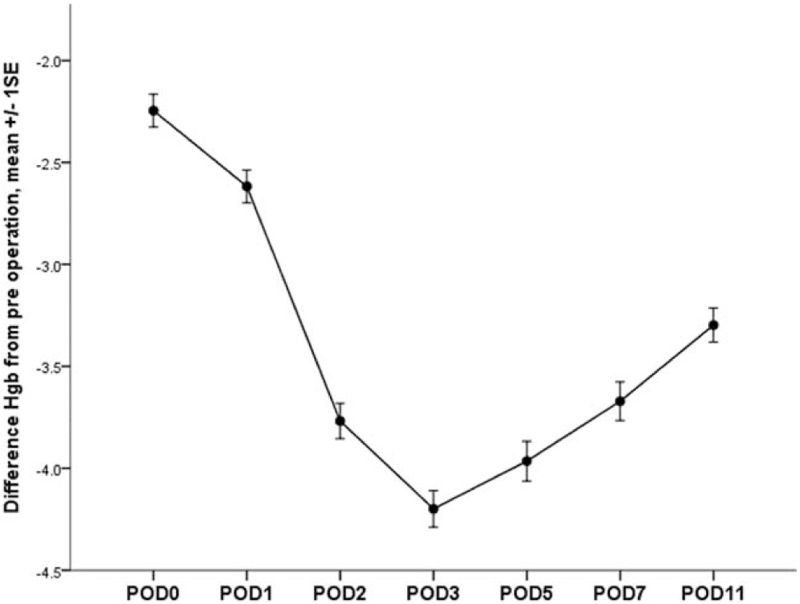
Mean difference from preoperative Hb level over time in patients who had undergone total knee arthroplasty (n = 177). POD = postoperative day.

Mean difference from preoperative Hb level over time are shown in Figure 2. Mean difference from preoperative Hb level were −2.24 ± 1.06 g/dL at the day of surgery, −2.61 ± 1.06 g/dL at POD 1, −3.76 ± 1.14 g/dL at POD 2, −4.19 ± 1.18 g/dL at POD 3, −3.96 ± 1.29 g/dL at POD 5, −3.67 ± 1.25 g/dL at POD 7, and −3.29 ± 1.11 g/dL at POD 11. The data showed that the timing of the Hb level was lowest at POD 3, starting to recover at POD 5. Targeting Hb level of 10 g/dL, 29 (16.4%) cases were recovered at POD 3, 38 (21.5%) cases at POD 5, 51 (28.8%) cases at POD 7, and 72 (40.7%) cases at POD 11. Based on preoperative Hb level, only 1 (0.6%) case showed recovery at POD 11.

### 3.2 The effects of tranexamic acid on the change of hemoglobin levels

In both groups, the lowest value of Hb was shown at POD 3, after which it tended to recover.

**Figure 3:**
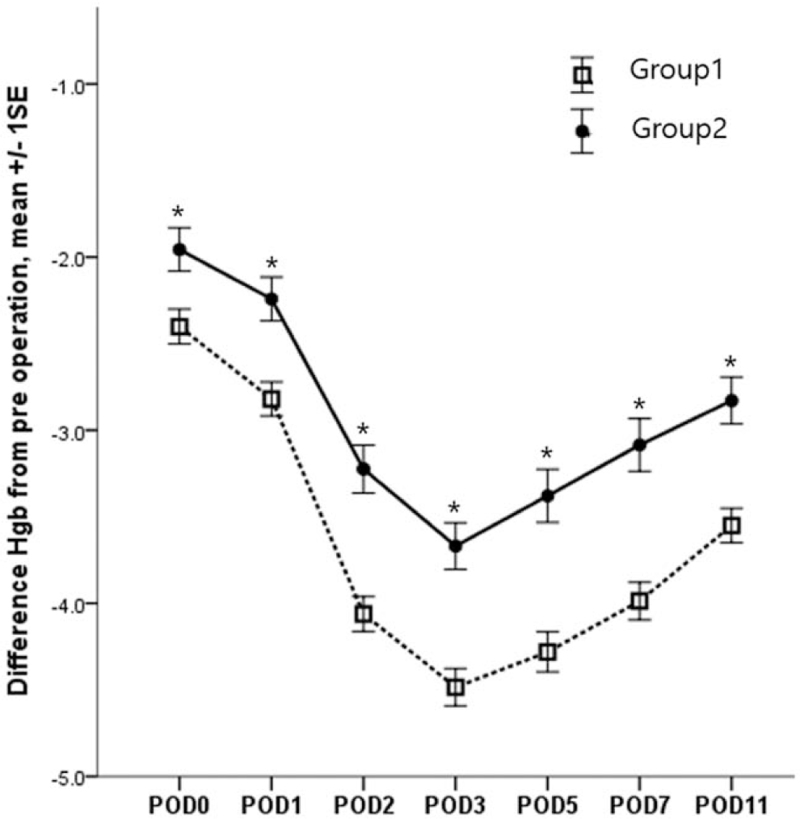
The same pattern of mean difference from preoperative Hb level in 2 groups. Group 1: navigated total knee arthroplasty without tranexamic acid. Group 2: total knee arthroplasty (manual method or navigation method) with tranexamic acid. ^∗^: *P* < .05. POD = postoperative day.

In group 1 (TKA without tranexamic acid injection), mean difference from preoperative Hb level were −2.40 ± 1.08 g/dL at the day of surgery, −2.82 ± 1.05 g/dL at POD 1, −4.06 ± 1.07 g/dL at POD 2, −4.48 ± 1.15 g/dL at POD 3, −4.28 ± 1.23 g/dL at POD 5, −3.98 ± 1.16 g/dL at POD 7, and −3.55 ± 1.05 g/dL at POD 11. The data showed that the timing of the Hb level was lowest at POD 3, starting to recover at POD 5 (Fig. 3). Targeting Hb level of 10 g/dL, 14 (12.2%) cases were recovered at POD 3, 19 (16.5%) cases at POD 5, 26 (22.6%) cases at POD 7, and 45 (39.1%) cases at POD 11. Based on preoperative Hb level, there was no case to recover at POD 11.

In group 2 (TKA with tranexamic acid injection), mean difference from preoperative Hb level were −1.95 ± 0.98 g/dL at the day of surgery, −2.24 ± 0.99 g/dL at POD 1, −3.22 ± 1.09 g/dL at POD 2, −3.66 ± 1.06 g/dL at POD 3, −3.37 ± 1.20 g/dL at POD 5, −3.08 ± 1.19 g/dL at POD 7, and −2.82 ± 1.06 g/dL at POD 11. The data showed that the timing of the Hb level was lowest at POD 3, starting to recover at POD 5 (Fig. 3). Based on 10 g/dL of Hb level, 15(24.2%) cases were recovered at POD 3, 19 (30.6%) cases at POD 5, 25 (40.3%) cases at POD 7, and 27 (43.5%) cases at POD 11. Based on preoperative Hb level, only 1 (1.6%) case showed recovery at POD 11.

In repeated measures ANOVA test, mean difference from preoperative Hb level showed a statistically significant difference between the 2 groups (*P* = .01) (Fig. 3). In post hoc test, the differences from preoperative Hb levels were statistically significantly lower at all measurements in group 2 (Table 2).

**Table 2:** *t* test to identify the difference in hemoglobin level compared to preoperation in 2 groups.

	Hb of group 1	Hb of group 2	Difference Hb of group 1	Difference Hb of group 2	*P* value
Pre OP	13.23 ± 1.20	12.86 ± 1.25			
POD 0	10.82 ± 1.15	10.91 ± 1.06	−2.40 ± 0.10	−1.96 ± 0.13	.008
POD 1	10.41 ± 1.10	10.62 ± 1.11	−2.82 ± 0.10	−2.24 ± 0.13	<.001
POD 2	9.17 ± 0.94	9.64 ± 1.13	−4.06 ± 0.10	−3.22 ± 1.09	<.001
POD 3	8.75 ± 0.91	9.19 ± 1.06	−4.48 ± 0.11	−3.67 ± 0.13	<.001
POD 5	8.95 ± 0.91	9.48 ± 1.03	−4.28 ± 0.12	−3.38 ± 0.15	<.001
POD 7	9.24 ± 0.92	9.78 ± 1.16	−3.99 ± 0.11	−3.09 ± 0.15	<.001
POD 11	9.68 ± 0.91	10.03 ± 1.10	−3.56 ± 0.10	−2.83 ± 0.14	<.001

Hb = hemoglobin, POD = Postoperative day. Group 1: navigated total knee arthroplasty without tranexamic acid injection. Group 2: total knee arthroplasty (manual method or navigation method) with tranexamic acid injection.

### 3.3 The effects of tranexamic acid on the change of hemoglobin levels according to gender

In both gender and group, the lowest value of Hb was shown at POD 3, after which it tended to recover.

In repeated measures ANOVA test, mean difference from preoperative Hb level showed a statistically significant difference between the 2 groups for both gender. In post hoc test, tranexamic group (Group 2) showed the statistically significantly lower differences from preoperative Hb levels at all measurements in both gender except for male in operation day (Table 3).

**Table 3:** *t* test to identify the difference in hemoglobin level compared to preoperation in 2 groups according to gender.

	Difference in Hb of group 1 (female, N = 80)	Difference in Hb of group 2 (female, N = 50)	*P* value	Difference in Hb of group 1 (male, N = 35)	Difference in Hb of group 2 (male, N = 12)	*P* value
POD 0	−2.34 ± 1.04	−1.88 ± 0.94	.01	−2.54 ± 1.17	−2.28 ± 1.16	.50
POD 1	−2.74 ± 1.06	−2.23 ± 0.96	.01	−3.01 ± 1.03	−2.31 ± 1.14	.05
POD 2	−3.89 ± 0.98	−3.15 ± 1.03	<.001	−4.47 ± 1.18	−3.52 ± 1.33	.03
POD 3	−4.31 ± 1.06	−3.64 ± 1.02	<.001	−4.89 ± 1.28	−3.80 ± 1.24	.01
POD 5	−4.04 ± 1.17	−3.28 ± 1.19	<.001	−4.84 ± 1.23	−3.78 ± 1.24	.01
POD 7	−3.76 ± 1.10	−2.96 ± 1.18	<.001	−4.50 ± 1.17	−3.60 ± 1.16	.03
POD 11	−3.31 ± 0.96	−2.78 ± 1.00	<.001	−4.11 ± 1.07	−3.03 ± 1.32	.01

Hb = hemoglobin, POD = Postoperative day. Group 1: navigated total knee arthroplasty without tranexamic acid injection. Group 2: total knee arthroplasty (manual method or navigation method) with tranexamic acid injection.

### 3.4 The difference in hemoglobin level for POD 4 weeks compared to preoperative in group 2 (TKA with tranexamic acid)

Among 62 cases of tranexamic acid injection in TKA, 58 cases were found to have useful Hb information at POD 4 weeks, with 52 (89.65%) recovering above the Hb level of 10 g/dL, and 8 (13.79%) above the preoperative Hb level.

## 4 Discussion

The important finding in our study is that, first, the patient showed the lowest Hb level at POD 3, which decreased by an average of 4.19 ± 1.18 g/dL compared to preoperative level, and later showed a tendency for the Hb level to gradually recover. Chen et al^[10]^ demonstrated that it takes approximately 4 days for the decline of Hb to reach its nadir and showed a tendency to recover afterwards. However, the study only described the trends over time, but did not show the difference in Hb level compared to preoperation. According to Pierson et al,^[11]^ the mean decline in the Hb level was 3.8 g/dL after TKA. However, the study evaluated the lowest Hb level, but could not reveal when the lowest level was.

Patients who had transfusion were excluded from the study, because the natural course of Hb after TKR would be manipulated. Also, patients who took antithrombotic agent were excluded, because it could increase the blood loss after TKA.^[12]^

Currently, staged bilateral TKA is conducted 1 or 2 weeks interval between the first and the second operation at many centers. Also, many centers frequently followed practice of the “10/30” rule. The “10/30” rule suggests that a patient receive a transfusion when the Hb level falls below 10 g/dL or the hematocrit falls below 30%.^[11,13]^ The anesthesiology department at our hospital also recommends that the patient's preoperative Hb level be at least 10 g/dL in TKA. Based on 10 g/dL of Hb level, 29 (16.4%) cases were recovered at POD 3, 38 (21.5%) cases at POD 5, 51 (28.8%) cases at POD 7, and 72 (40.7%) cases at POD 11. Our study shows that more than half of patients underwent TKA with Hb levels below 10 g/dL. Eventually, in staged bilateral TKA, many strategies must be taken to reduce the need for blood transfusion.

The second important finding is that, regardless of the injection of tranexamic acid, it takes approximately 3 days for the decline of Hb to reach its nadir and gradually recover afterwards by the same tendency. Also, in a group with tranexamic acid injection, the differences from preoperative Hb levels at all measurements were significantly lower. It proved that the use of tranexamic acid is effective in reducing the decline of Hb after TKA such as previous studies.^[14,15]^

The third important finding is that out of 62 cases of tranexamic acid injection, a total of 58 cases were found to have meaning Hb information at POD 4 week, with 52 (89.65%) showing a recovery of 10 g/dL in Hb levels and 8 (13.79%) showing a recovery of the preoperative Hb level.

The present study has several limitations. First, this study is retrospective design, second, since no samples were collected at POD 4, it is not clear whether the lowest Hb level is actually at POD 3 or POD 4. Third, 2 different surgeon performed the TKR. Because of this, there could be an error in the result between 2 surgeons. However, what this study revealed was that the changes in Hb showed the same pattern regardless of the operator.

## 5 Conclusion

When deciding whether to transfuse after TKA, it should be noted that the patient tends to show the lowest Hb level POD 3. Also, the authors emphasize that tranexamic acid injection in the joint is an effective method to reduce the loss of Hb after TKA.

## Author contributions

**Conceptualization:** Myung Rae Cho, Won Kee Choi.

**Data curation:** Chung Mu Jun, Suk Kyoon Song, Won Kee Choi.

**Formal analysis:** Suk Kyoon Song, Won Kee Choi.

**Investigation:** Suk Kyoon Song, Won Kee Choi.

**Methodology:** Won Kee Choi.

**Supervision:** Won Kee Choi.

**Validation:** Chung Mu Jun, Won Kee Choi.

**Visualization:** Suk Kyoon Song.

**Writing – original draft:** Myung Rae Cho, Suk Kyoon Song.

**Writing – review & editing:** Myung Rae Cho, Chung Mu Jun, Won Kee Choi.
